# Individual Differences in Holistic Processing Predict the Own-Race Advantage in Recognition Memory

**DOI:** 10.1371/journal.pone.0058253

**Published:** 2013-04-10

**Authors:** Joseph DeGutis, Rogelio J. Mercado, Jeremy Wilmer, Andrew Rosenblatt

**Affiliations:** 1 Geriatric Research Education and Clinical Center (GRECC), Boston Division VA Healthcare System, Jamaica Plain, Massachusetts, United States of America; 2 Vision Sciences Laboratory, Department of Psychology, Harvard University, Cambridge, Massachusetts, United States of America; 3 Department of Psychology, Temple University, Philadelphia, Pennsylvania, United States of America; 4 Department of Psychology, Wellesley College, Wellesley, Massachusetts, United States of America; National Institute of Mental Health, United States of America

## Abstract

Individuals are consistently better at recognizing own-race faces compared to other-race faces (other-race effect, ORE). One popular hypothesis is that this recognition memory ORE is caused by differential own- and other-race holistic processing, the simultaneous integration of part and configural face information into a coherent whole. Holistic processing may create a more rich, detailed memory representation of own-race faces compared to other-race faces. Despite several studies showing that own-race faces are processed more holistically than other-race faces, studies have yet to link the holistic processing ORE and the recognition memory ORE. In the current study, we sought to use a more valid method of analyzing individual differences in holistic processing by using regression to statistically remove the influence of the control condition (part trials in the part-whole task) from the condition of interest (whole trials in the part-whole task). We also employed regression to separately examine the two components of the ORE: own-race advantage (regressing other-race from own-race performance) and other-race decrement (regressing own-race from other-race performance). First, we demonstrated that own-race faces were processed more holistically than other-race faces, particularly the eye region. Notably, using regression, we showed a significant association between the own-race advantage in recognition memory and the own-race advantage in holistic processing and that these associations were weaker when examining the other-race decrement. We also demonstrated that performance on own- and other-race faces across all of our tasks was highly correlated, suggesting that the differences we found between own- and other-race faces are quantitative rather than qualitative. Together, this suggests that own- and other-race faces recruit largely similar mechanisms, that own-race faces more thoroughly engage holistic processing, and that this greater engagement of holistic processing is significantly associated with the own-race advantage in recognition memory.

## Introduction

Human visual memory is remarkable in its capacity to discriminate between thousands of previously seen faces. Despite this expertise, people are generally better at remembering and individuating own-race faces compared to other-race faces, a phenomenon termed the other-race effect (ORE; for a review see [1]). The ORE is among the most robust findings in the face recognition literature, and has been replicated across many cultures (for a review see [2]). Although it first emerges in infancy at around six months of age [3], the ORE is malleable in both children and adults through increased other-race individuation experiences [4] and structured individuation training with other-race faces [5].

Current dominant models of the ORE (perceptual expertise and socio-cognitive) emphasize that own-race faces, compared to other-race faces, more fully engage specialized holistic face processing mechanisms [6], [7], [8]. A popular definition of holistic face processing is the simultaneous integration of feature, spacing, and face contour information into a single coherent representation [9], [10]. Perceptual expertise models suggest that prolonged experience with discrimination and individuation of own-race faces creates a rich holistic representation of the facial structure of one's own race; in contrast, less experience individuating other-race faces results in a relatively impoverished and less holistic representation of other-race facial structures [11]. The critical role of visual experience in the ORE is supported by developmental studies. For example, Kelly and colleagues found that 3-month-old Western European infants can discriminate faces within four different racial groups (faces of their own racial group, sub-Saharan Africans, Middle Eastern, and Chinese faces), whereas 9-month-old infants can only discriminate own-race faces [3]. Recent studies have emphasized the importance of active face individuation experience, rather than passive exposure, to the development of the ORE [12], [13]. Additionally, studies have shown that individuation training with other-race faces, though not categorization training, can enhance recognition of other-race faces [5], [14].

In contrast to expertise models, socio-cognitive models emphasize that social and motivational factors can both produce and diminish the ORE. According to the individuation/categorization model, individuals attend to *identity*-diagnostic characteristics in own-race/in-group faces (e.g., configural information in the eye region) and engage holistic face processing mechanisms [15]. In contrast, individuals attend more to *category*-diagnostic features of other-race/out-group faces such as surface properties of faces (e.g., skin tone and specific features). This leads to poorer recognition of other-race/out-group faces, but potentially more efficient classification ability. Evidence supporting socio-cognitive models is from Bernstein, Young, and Hugenberg [16], which suggests that social categorization alone is sufficient to elicit differential face recognition performance. Using Caucasian Miami University students and Caucasian face stimuli, they demonstrated better memory for faces arbitrarily labeled from Miami University compared to those labeled as being from Marshall University, a rival school. This suggests that the ORE may be a special case of more general in-group/out-group biases. Additional support for a social motivational account comes from studies that abolish the other-race effect by explicitly informing participants about the other-race effect and providing instruction to individuate other-race faces [17], [18]. This suggests that individuals have latent individuation and holistic processing expertise with other-race faces that they do not typically utilize because of social and motivational factors.

The idea that greater holistic perceptual processing of own-race compared to other-race faces is a strong determinant of the other-race effect in recognition memory is important to both expertise and socio-cognitive theories. Developing a holistic representation through repeatedly individuating own-race faces is crucial to expertise models, whereas motivation/attention enhancing holistic processing and improving individuation of own-race faces is crucial to socio-cognitive models. If there is not an association between differential own- and other-race holistic processing and the recognition memory ORE, these current models would have to be substantially revised. A lack of association would suggest that other factors, such as differential parts-based processing, might be more important than holistic processing to the recognition memory ORE. A lack of association could also suggest that memory consolidation mechanisms are more important to the recognition memory ORE than perceptual processing. Demonstrating a strong association between differential holistic processing and the recognition memory ORE would help to reinforce the current theoretical directions of models of the ORE and would further stimulate investigations in this area.

Despite the importance of the holistic processing/recognition memory ORE association, few studies have explicitly tested this association. A recent ERP study provides some indirect support by demonstrating a significant correlation between the size of the recognition memory ORE and the N170 difference during own- and other-race face encoding [19], an ERP component thought to reflect configural and holistic processing [20]. Unfortunately, the few behavioral studies who have directly tested this association failed to find a significant correlation (Michel a [6]: Asian participants, r = −.06, n.s., Caucasian participants, r = −.06, n.s.; Michel b [7]: Asian participants, r = −.18, n.s., Caucasian participants, r = .15, n.s.). Hancock and Rhodes (2008), using the face inversion effect as a measure of holistic processing, are to our knowledge the only report to successfully demonstrate a significant relationship between a behavioral measure of holistic processing and the recognition ORE [21]. However, they used the same trials to calculate the holistic processing effect ([upright own-race minus inverted own-race] minus [upright other-race minus inverted other-race]) as they did to calculate the recognition ORE (upright own-race minus upright other-race). This is problematic because non-independent measures such as these commonly produce spurious correlations [22], [23]. Thus, the crucial link between the holistic processing ORE and the recognition memory ORE remains to be convincingly demonstrated.

One possibility is that there is a significant association between the ORE in recognition memory and the ORE in holistic processing, but that this association has been obscured by the manner in which holistic processing measures and ORE measures have been calculated. Measures of holistic face processing (e.g., part-whole task) are routinely calculated by subtracting a control condition that does not engage holistic processing from a condition that does (e.g., subtracting part from whole trials in the part-whole task). The problem with this subtraction approach is that the resulting measure is yoked to the control condition, thus producing measures of holistic processing confounded by the control condition. This situation is typically not intended by the researcher [24]. An alternative that more validly isolates holistic processing is to, across individuals, regress the control condition from the condition of interest. Compared to the subtraction approach, when using the regression approach to measure holistic processing in the part-whole and composite tasks, DeGutis and colleagues found stronger correlations amongst holistic processing measures (demonstrating construct validity of holistic processing) and stronger correlations between these separate holistic processing measures and face recognition ability (providing converging evidence for the holistic processing/recognition memory link) [24]. In the context of the other-race effect, using a regression approach to measure holistic processing may better characterize the holistic processing ORE/recognition memory ORE association.

**Figure 1:**
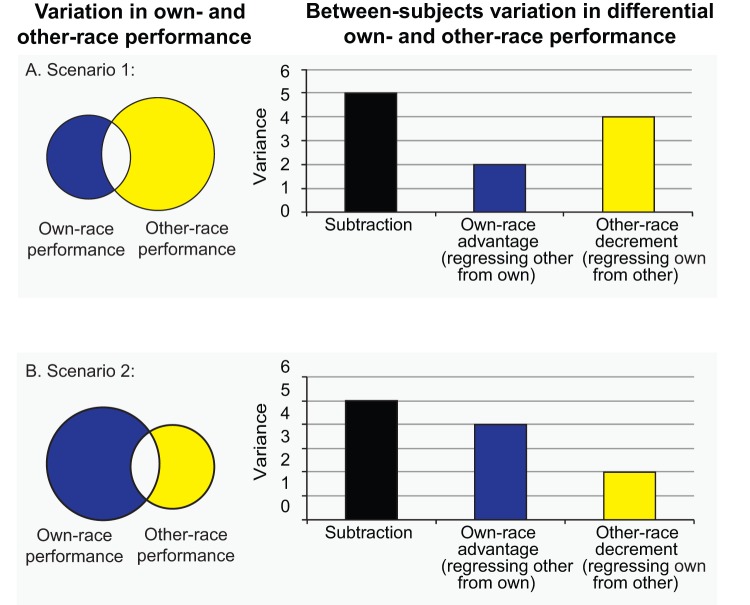
Subtraction vs. regression measures of the other-race for scenarios of unequal variance. The circles in the Venn diagrams on the left represent individual variation in own- and other-race performance and the size of the circles indicates the amount of individual variation. The blue area represents individual variation specific to own-race faces (own-race advantage: other-race performance regressed from own-race) and the yellow area represents individual variation specific to other-race faces (other-race decrement: own-race performance regressed from other-race). The bar graphs on the right represent the amount of variance in subtraction measures as well as own-race advantage and other-race decrement regression measures. The point of this demonstration is that subtraction obscures the source of the variation in its component conditions and provides the same variance measure for the two scenarios whereas regression is able to isolate the source of the variance.

Another important issue at the core of characterizing the link between the holistic processing ORE and recognition memory ORE is how to best compare own- and other-race face performance. Traditionally, the other-race effect has been calculated by subtracting other-race performance from own-race performance. The theoretical stance behind this calculation is that better own-race performance and worse other-race face performance equally and oppositely contribute to the other-race effect. Despite the intuitive appeal of this, there are practical and theoretical reasons to separately measure the boost one gets when processing own-race faces while controlling for other-race performance (i.e., own-race advantage, see blue area in Figure 1 and Methods for a more detailed theoretical explanation), and the performance decrement one gets when processing other-race faces while controlling for own-race performance (i.e., other-race disadvantage, see yellow area in Figure 1 and Methods) [25]. First, in practice, across a group of subjects the contribution of the own-race advantage and other-race decrement to the traditional ORE is rarely perfectly equivalent. This could result from restriction of range issues in either measure or rather because one measure has more individual variation than the other because of theoretically important reasons (e.g., individuals may perform somewhat similarly on other-race faces because they engage similar race-general processing mechanisms). Figure 1b and c illustrates scenarios when there is more variance in the own-race advantage compared to the other-race decrement and vice versa. Traditional subtraction measures of the ORE obscure the contributions of its constituents and cannot distinguish between these different scenarios. In contrast, using regression to compare own- and other-race processing allows one to isolate individual variation in the own-race advantage separately from individual variation in the other-race decrement (Figure 1a,b,c), enabling one to test for associations in a more specific manner than the subtraction approach.

In addition to providing a better understanding of the relative contribution of the separate components when their contribution is not equivalent, there may also be theoretical reasons for separately measuring the components of the traditional ORE. For example, several researchers have conceptualized the ORE as an ‘own-race bias’, ‘own-race advantage’, or ‘same-race advantage [1], [26], [27], [28], suggesting that the boost in own-race performance when controlling for other-race performance is of particular interest. Conversely, according to Rodin’s cognitive disregard model, the ‘turning off’ of certain processes when in the presence of other-race faces may reflect a distinct and active process [25]. Even if one still believes that the ORE reflects both the own-race performance advantage combined with the other-race performance decrement, examining these effects separately could help provide additional theoretically important information and may ultimately lead to refining models of the ORE. For these reasons, we examined the ORE in both the traditional manner (subtracting other-race from own-race performance), as well as by separately examining the own-race advantage (regressing other-race face performance from own-race face performance) and other-race decrement (regressing own-race face performance from other-race face performance).

In particular, to examine the link between differential holistic processing and differential recognition memory for own- and other-race faces, we chose previously validated measures of holistic face processing (Caucasian and Asian versions of the classic part-whole task [8], [29]), and face recognition ability (Caucasian and Asian versions of the Cambridge Face Memory Test [30], [31]). To calculate holistic processing, we used the commonly used subtraction approach as well as previously validated regression-based approach [24]. To quantify differential processing of own- and other-race faces, we also used the traditional subtraction approach and novel regression-based measures that separately quantify the own-race advantage and other-race decrement. This allowed us to sufficiently assess whether any component of differential holistic processing is related to any component of differential recognition memory between own- and other-race faces. Finally, we sought to understand whether own- and other-race faces are processed using similar or different mechanisms. This would provide evidence of whether differences in own- and other-race face processing is qualitative or quantitative. To investigate this, we correlated own- and other-race part, holistic, and recognition memory performance, and we compared part/holistic vs. CFMT correlations across own- and other-race faces.

## Methods

### Participants

53 individuals (24 males) with a mean age of 24.91 years (SD  = 4.83) participated in the study for compensation ($10/hour). Participants were recruited from a community message board and included local university students as well as other community members of the greater Boston area. All participants self-reported as having solely a Caucasian ethnicity. The ethics of this study, in addition to the written informed consent forms obtained from all participants, were approved by and in compliance with the Institutional Review Board (IRB) of the VA Boston Healthcare System. Participants were tested at the VA Medical Center in Boston or at the Harvard University Vision Sciences Laboratory in Cambridge, MA. All participants had normal or correct-to-normal vision and none reported a history of neurological psychiatric illness, or difficulty in remembering faces. Because the current study was part of a larger experiment investigating cognitive training-related changes in face processing, participants performed the questionnaire and tasks in the following fixed order: other-race effect contact survey, Cambridge Face Memory Test (CFMT) Caucasian, CFMT Asian, part-whole task (PW) Caucasian, and PW Asian. Performing these tasks in a fixed order decreases the between-subjects variance attributable to different test orders but leaves open the possibility of order effects (see discussion).

### Other-race effect contact survey

Contact with Asian and Caucasian individuals was measured using a questionnaire developed by Hancock and Rhodes (2008) [21], modified by replacing the term “Chinese” with “Asian”. Of 14 statement items, seven indicated contact with Caucasians and seven with Asians. Statements were identical in wording except for the race term. Examples include: “I socialize a lot with (Asian/Caucasian) people,” and “I generally only interact with (Asian/Caucasian) people.” Responses were measured on a 6-point scale (1 =  very strongly disagree; 2 =  strongly disagree; 3 =  disagree; 4 =  agree; 5 =  strongly agree; 6 =  very strongly agree). Hancock and Rhodes reported high internal consistency in both Caucasian participants (Cronbach's α = .92, own-race faces; α = .82, other-race) and Chinese participants (α = .89, own-race; α = .94, other-race) [21]. We similarly found high internal consistency in our Caucasian participants (α = .69, own-race faces; α = .79, other-race).

### Cambridge Face Memory Tests

We chose the Cambridge Face Memory Test (CFMT) as our measure of face recognition memory because it has high reliability and validity, and because it has validated Asian and Caucasian versions. In particular, the internal reliability of the original Caucasian version in published studies ranges from .86 to .89 (α = .89 [32]; α = .86 [30]; α = .88 [33]) and its test-retest reliability is .70 [32]. Its high validity is shown by its face specificity: it correlates highly with other face-related measures (naming of famous faces: r = .70 [34], and r = .51 [32]; face perception: r = .60 [33]), yet correlates more modestly with measures of non-face visual memory (r = .26) and verbal memory (r = .17) [32]. The Asian CFMT also yields high internal reliability for both Asian participants (r = .90) and Caucasian participants (r = .89) [31].

### Stimuli and Procedure

Participants learned to recognize six target faces, excluding non-facial cues that could be used for differentiation (e.g., hair, see [30], [31]), and were tested in progressively more difficult stages. During the introductory phase, a target face was presented from three different views (front, right profile, left profile) for 3 seconds per view. After this, participants were presented with 3 three-alternative forced-choice trials, where they identified the target face among two foils, with one trial for each of the three views. The process was repeated for the remaining 5 faces, resulting in 18 total trials. Next, participants studied these same 6 target faces shown all at once for 20 seconds. Following this study period, participants were tested on 30 trials where they identified a target face among 2 foils from novel views and with changes in lighting. Participants then received 20 more seconds to study the same 6 target faces. The remaining 24 trials were the most difficult and presented faces with novel views, lighting changes, and the addition of visual noise.

Each participant completed the original CFMT that used Caucasian faces and an identical format that uses Asian faces (created by and used with permission of Jia Liu of Beijing Normal University [31]).

### Part-Whole Tasks

We used the part-whole task (PW) because it is a widely accepted measure of holistic processing and because it includes Asian and Caucasian stimuli that have previously been shown to demonstrate a significant participant race x stimulus race x part/whole (i.e., holistic processing) interaction (used with permission from Jim Tanaka, University of Victoria [8]). The part-whole task assesses how much subjects integrate individual facial features into the whole face context. In particular, after encoding a target face (e.g., Roger's face), subjects demonstrate an advantage for discriminating a feature change (e.g., discriminating Roger's nose from Ken's nose) when features are shown within the context of the target face (whole trials) compared to when discriminating features are shown in isolation (part trials). Our logic was that between-subjects variation in part trials primarily reflects general visual perception as well as face part processing abilities, whereas between-subjects variation in whole trials reflects general visual perception, face part processing, and holistic face processing abilities. Though some strong versions of the definition of holistic face processing suggest that, when shown a whole face (such as in whole trials), there is little or no part processing or part representation [35]. We and others suggest that there is some explicit part processing/representation and that holistic face processing further facilitates part recognition [36]. In support of features having some explicit representation, Reinitz and colleagues demonstrate that subjects will often claim to have previously seen a new face if they have previously seen faces containing its component features [37]. Thus, we reasoned that regressing part trial performance from whole trials would provide a relatively pure measure of holistic face processing (see analysis section below for further details).

### Stimuli & Procedure

Target faces were created using either a Caucasian male, Caucasian female, Asian male, or Asian female face template that included the hair and face outline. For each template, 6 target faces were created, each with a different nose, mouth, and pair of eyes inserted into the template (for an example, see [8]). Therefore, each target face was unique and did not share a feature with another target face. Foils for each target face were created by switching one of the three facial components (eyes, nose, or mouth) with that of a different target face.

For each trial in the PW, participants were initially presented with a central fixation for 500 ms. A whole target face was then centrally presented for 1000 ms, followed by a mask (scrambled face) for 500 ms. Next, participants were presented with either a whole trial, in which one stimulus was the target face and the other a foil, or a part trial, in which only a given isolated feature (eyes, nose, or mouth) from both the target and foil face were presented. On whole trials (50%), participants were asked to indicate which whole face matched the target face, and for part trials (50%), participants were asked to indicate which isolated face feature matched the target face. For both part and whole trials, the stimuli were presented side by side and remained on the screen until the participants made a response of “1” for the left stimulus or “2” for the right stimulus. There was a single session of 72 trials for each gender (36 parts trials and 36 whole trials), with equal numbers testing eyes, nose, and mouth, with gender being blocked and counterbalanced.

### Individual Differences Analyses: Computing the Part-whole Holistic Advantage

For the PW, we calculated holistic processing scores using both subtraction and regression. Subtraction has been used in prior studies [38], [39], while regression has been demonstrated to create a measure of holistic processing that more strongly correlates with another widely accepted measure of holistic processing (composite task) and is substantially more correlated with face recognition ability (regression: r = .46; subtraction: r = .26 [24]). Subtraction measures were produced by simply subtracting part trial performance from whole trial performance. Because the logic behind the part-whole task is that only the whole trials include the process of interest (i.e., holistic processing) while part trials do not, regression scores, which isolate individual differences specific to whole trials, were our primary measure of interest. To calculate regression scores, we examined whole trial performance while statistically controlling for part trial performance (for a more in-depth description and illustration of this procedure, see [24]). Briefly, regression scores measure how a given individual's whole trial performance compares to the typical person with the same part trial performance. A typical person's whole trial performance for any part trial performance is represented with a least squares regression line (observed part trial performance predicting observed whole trial performance) and the distance of each individual's whole trial performance above or below this line represents how each individual's whole performance deviates from the best estimate of the mean whole performance for all other individuals with the same part score. In this way, a regression measure is created that statistically equates all individuals' part scores and measures that portion of their whole performance that is not accounted for by their part performance.

### Individual Differences Analyses: Computing Other-race Effects, Own-race Advantages and Other-race Decrements

**Figure 2:**
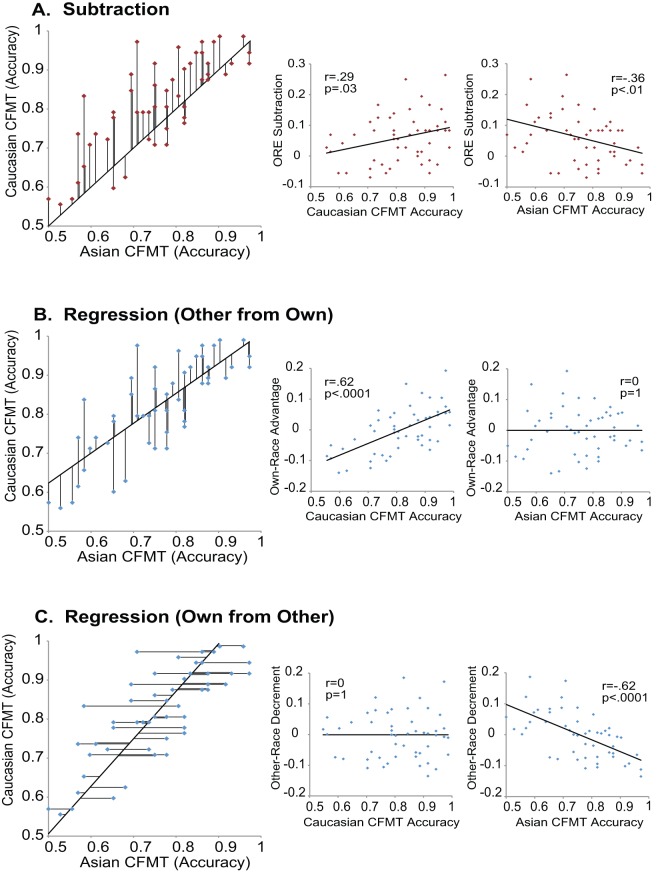
Other-race effect measures for the Cambridge Face Memory Test and their correlations with their constituent conditions. Individual differences in the other-race effect were calculated three ways: A) subtraction, where other-race performance is subtracted from own-race performance to produce a difference score (top row, red plots, each difference score is indicated with a vertical black line), B) regressing other- from own-race performance to produce own-race advantage residuals (second row, blue plots, each regression residual is indicated with a vertical black line), or C) regressing own- from other-race performance to produce other-race decrement residuals (third row, blue plots, each regression residual is indicate with a horizontal black line). As can be seen in the smaller graphs on the right, the subtraction approach creates a measure that is both positively correlated with own-race performance and negatively correlated with other-race performance. In contrast, the own-race advantage residuals are correlated with own-race performance but not with other-race performance, whereas other-race decrement residuals are correlated with other-race performance but not with own-race performance.

To calculate differential own- and other-race processing for the part-whole and CFMT, we employed the traditionally used subtraction approach [6], [7], [18], as well as the regression approach to separately examine the own-race advantage and other-race decrement (see Figure 2, a,b,c). The difference between the use of subtraction to measure the holistic advantage in the part-whole versus the ORE is that subtraction does not capture the logic of the part-whole (i.e., part trials do not engage holistic processing whereas whole trials do) whereas for the ORE, subtraction could approximate the logic of the ORE that many researchers advocate, albeit tacitly (i.e., that the ORE arises from better performance with own-race faces combined with worse performance with other-race faces). That said, examining the components of the ORE separately using regression could help clarify their unique contributions to the ORE. As can be seen from positive correlations between subtraction measures and the own-race recognition memory and negative correlations between subtraction measures and other-race recognition memory in Figure 2a, subtraction measures of the ORE are yoked to their two component scores in a way that obscures the relative contribution of each component to their variation (see also Figure 1). A low subtraction score could result entirely from exceptional performance with other-race faces, entirely from poor performance with own-race faces, or from some combination of the two. Likewise, a correlation between ORE subtraction measures (ORE in recognition memory vs. ORE in holistic processing) could result entirely from variation in the other-race face conditions, entirely from variation in the own-race face conditions, or from some combination of the two. Or, more in line with the current literature, a lack of a correlation between ORE subtraction scores could result from measures of one component having a different effect than measures of the other component, potentially obscuring a significant relationship.

Regression scores, in contrast to subtraction scores, can isolate variation in one component at a time, with the variation in the other component statistically removed (see Figure 2b and c). For example, as is exemplified in Figure 2b, regression can measure the own-race advantage as the variation left over in own-race face performance after the variation it shares with other-race performance is removed. Regression accomplishes this by essentially asking how a given individual's own-race performance compares to the typical person with the same other-race performance. In this way, a regression measure is created that statistically equates all individuals' other-race effect scores and measures that portion of their own-race performance that is not accounted for by their other-race performance. The same procedure can be performed to measure the other-race performance decrement by calculating the variation left over in other-race face performance after the variation it shares with own-race performance is removed (see Figure 2c).

Though it is clear that regression can statistically separate the ORE into its own-race advantage and other-race decrement components, what are the theoretical implications of this? The key difference between these components is their emphasis on own- or other-race faces as the critical measure. For the own-race advantage component, other-race faces are treated as one's baseline face abilities and variance in these trials are treated as noise, whereas the differential boost in own-race face performance is treated as the measurement of interest. In other words, the theory of the own-race advantage suggests that there are some basic face processing mechanisms that are recruited by faces of any ethnicity and the effect of interest is how much, across individuals, own-race faces differentially elicit additional processing mechanisms. In contrast, the other-race decrement component treats own-race face performance as the baseline and individual variance in these trials as noise while treating the decrement in other-race performance as the measure of interest. This suggests that everyone is at his or her maximum performance for own-race faces and that the effect of interest is the degree to which individuals' performance differentially worsens with other-race faces. Thus, being able to separately explore the own-race advantage and other-race decrement may clarify the relative contributions of own-race and other-race processing to the other-race effect.

## Results

### Participant demographics and race contact

**Figure 3:**
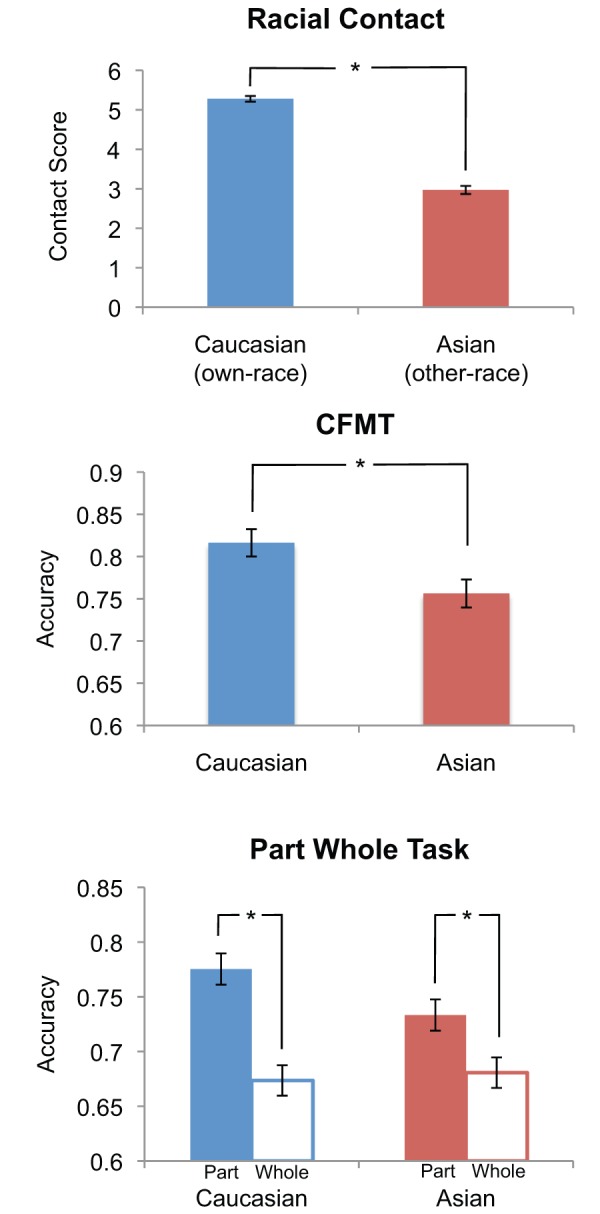
Caucasian and Asian Contact Questionnaire Scores and Performance on the Caucasian and Asian CFMT and Part-Whole Tasks. A) The Caucasian participants reported significantly more contact with Caucasian than Asian individuals. B) Participants also showed significantly better recognition memory accuracy on the Caucasian compared to the Asian CFMT and C) a larger holistic advantage on the Caucasian compared to Asian part-whole task (though both Caucasian and Asian tasks demonstrated a significant holistic advantage). Error bars indicate the standard error of the mean and * indicates a significant effect p<.05.

As shown in Figure 3a, the surveys demonstrated that our Caucasian participants reported significantly more contact with Caucasian than Asian people (Caucasian contact M = 5.28, SD = .52; Asian contact M = 2.97, SD = .75, t(52) = 17.38, p<.001). This Asian contact score is slightly less than that of Caucasian participants reported by Hancock and Rhodes [21] (M = 3.5, SD = .9, note: they did not report a Caucasian contact score).

### Group Performance Differences between Own- and Other-race Faces

We next sought to confirm that our CFMT and PW results are in line with previous reports and that they demonstrate significant own- vs. other-race differences. As can be seen in Figure 3b, subjects performed significantly better on the CFMT Caucasian (CFMT, M = 81.6% correct, SD = 11.8) than the CFMT Asian (M = 75.6%, SD = 12.1) (t(52) = 5.60, p<.0001). This is somewhat smaller of an effect (though not significantly different) than a recent report of Caucasian participants by McKone and colleagues (Caucasian CFMT M = 76.0%, SD = 11.7; Asian CFMT M = 66.0%, SD = 14.4) [31] and is likely due to their small sample size (their N was only 20) rather than demographic differences (Canberra, Australia has as large or larger Asian population compared to Boston).

As can be seen in Figure 3c, the part-whole holistic advantage was larger for own-race (Caucasian) faces than other-race (Asian) faces (significant stimulus race x part/whole interaction, F(1,52) = 13.10, p<.001), though both Caucasian and Asian faces showed significant holistic advantages (Caucasian faces: (t(52) = 10.43, p<.0001; Asian faces (t(52) = 4.39, p<.0001). Breaking this effect down into the separate whole and part trials, participants showed better performance on Caucasian whole trials compared to Asian whole trials (t(52) = 4.20, p<.0005) but no differences between Caucasian and Asian part trials (t(52) = .66, p = .51). These results are very similar to that reported by Tanaka and colleagues [8], who used an identical procedure and stimuli, except that Caucasian participants in that study did not show a significant holistic advantage for Asian faces.

**Figure 4:**
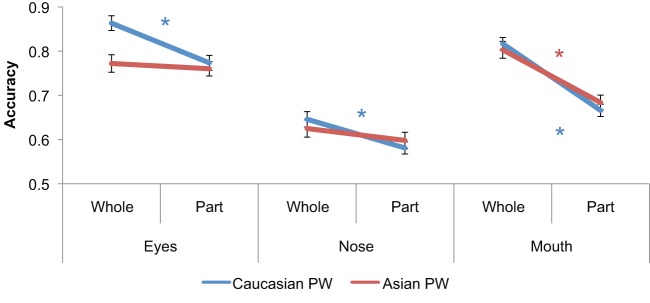
Part-Whole Performance Broken Down by Eyes, Nose, and Mouth. Participants demonstrated nearly identical patterns of accuracy on Caucasian and Asian nose and mouth trials, but were significantly worse on Asian eye whole trials compared to Caucasian eye whole trials. Error bars indicate the standard error of the mean and * indicates a significant part vs. whole effect p<.05.

Breaking down the PW by facial feature, as is shown in Figure 4, revealed a significant difference in holistic processing between own- and other-race faces for the eye region (significant stimulus race x part/whole interaction, F(1,52) = 13.27, p<.001), but not for the nose (F(1,52) = 1.94, p = .17), and revealed only a trend for the mouth region (F(1,52) = 2.78, p<.10). In particular, for the eye region, participants showed a robust holistic advantage for Caucasian faces (t(52) = 6.42, p<.0001), but no evidence of holistic processing for Asian faces (t(52) = .69, p>.49). For the mouth region, participants showed a robust holistic advantage for both Caucasian (t(52) = 9.84, p<.0001) and Asian faces (t(52) = 6.44, p<.0001). To summarize the results so far, when comparing Asian and Caucasian faces, we demonstrate robust differences in recognition memory accuracy and holistic face processing, replicating previous reports. We further demonstrate that greater holistic face processing for own- compared to other-race faces is specific to the eye region.

### Individual Differences Analyses

#### Reliability of Face Recognition Ability and Measures of Holistic Face Processing

To better evaluate our individual differences correlations below, we first calculated the internal reliability of each measure (for the rationale and methods, see File S1). This is crucial as it provides a measure of the upper bounds or maximum possible correlations between measures and provides context for interpreting the empirically observed correlations.

As can be seen in Table 1, our analyses showed Caucasian and Asian CFMTs to have very high reliability (Caucasian CFMT: λ2 = .90; Asian CFMT: λ2 = .88). Additionally, we found robust reliabilities for the CFMT ORE (λ2 = .48), CFMT own-race advantage (λ2 = .52), and CFMT other-race decrement (λ2 = .54). For the PW, the separate part and whole conditions showed fairly good reliability (all λ2′s >.5, see Table 1), though the holistic advantage difference scores and residual scores were overall much less reliable (Asian holistic advantage regression: λ2 = .48; subtraction: λ2 = .38; Caucasian holistic advantage regression: λ2 = .33; subtraction: λ2 = .07). The PW ORE and PW other-race decrement reliabilities were near zero (λ2 = −.01, λ2 = −.14, respectively), whereas the own-race advantage demonstrated a modest λ2 reliability of .25. The low reliability of the PW ORE makes it mathematically challenging to achieve a significant correlation with another measure and may explain why previous attempts failed to find a significant link between the holistic processing ORE and recognition memory ORE [6], [7].

**Table 1:** Reliabilities for Cambridge Face Memory and Part-Whole Measures.

	Λ2 (α)
**CFMT Caucasian**	.9 (.88)
**CFMT Asian**	.88 (.86)
**PW Caucasian**	
Whole	.73 (.68)
Part	.5 (.42)
HP Subtraction	.07 (−.09)
HP Regression	.33 (.21)
**PW Asian**	
Whole	.79 (.76)
Part	.76 (.42)
HP Subtraction	.38 (−.13)
HP Regression	.48 (.20)
**ORE Subtraction**	
Part Trials	.00 (−.57)
HP Subtraction	−.01 (−.45)
HP Regression	.05 (−.28)
CFMT	.48 (.39)
**Own-Race Advantage**	
Part Trials	.27 (−.34)
HP Subtraction	−.02 (−.22)
HP Regression	.25 (−.07)
CFMT	.52 (.46)
**Other-Race Decrement**	
Part Trials	.01 (−.34)
HP Subtraction	.29 (−.26)
HP Regression	−.14 (−.05)
CFMT	.54 (.44)

#### Holistic Processing Own-race Advantage Predicts the Own-race Advantage in Recognition Memory

The main goal of the current study was to determine whether aspects of the holistic processing other-race effect are significantly associated with aspects of the recognition memory other-race effect. These results are summarized in Table 2. When employing the subtraction approach to measure both holistic processing (whole trials minus part trials) and the recognition memory ORE (own-race minus other-race), we found a weak and non-significant relationship between the holistic processing ORE and recognition memory ORE (r = .10, p = .49), replicating previous studies (e.g., [6], [7]). The zero reliability of the subtraction-based PW ORE (λ2 = −.01) makes this a likely outcome even if there were some theoretical relationship. We additionally used regression to calculate holistic processing (regressing part performance from whole performance) while using subtraction to calculate the ORE. This only marginally improved the holistic processing/recognition memory ORE correlation (r = .15, p = .27).

**Table 2:** Correlations Between Differential Own- and Other-race Recognition Memory and Holistic Processing.

	CFMT ORE Subtraction	CFMT Own-Race Advantage	CFMT Other-Race Decrement
**ORE Subtraction**			
HP Subtraction	.10	.08	−.10
HP Regression	.15	.11	−.10
Part Trials	.10	.06	−.13
**Own-Race Advantage**			
HP Subtraction	.18	.24	−.11
HP Regression	.18	**.27** *	−.09
Part Trials	−.01	.10	.10
**Other-Race Decrement**			
HP Subtraction	.00	.06	.06
HP Regression	−.09	.03	.18
Part Trials	−.11	−.06	.15

* bold indicates a significant correlation p<.05.

**Figure 5:**
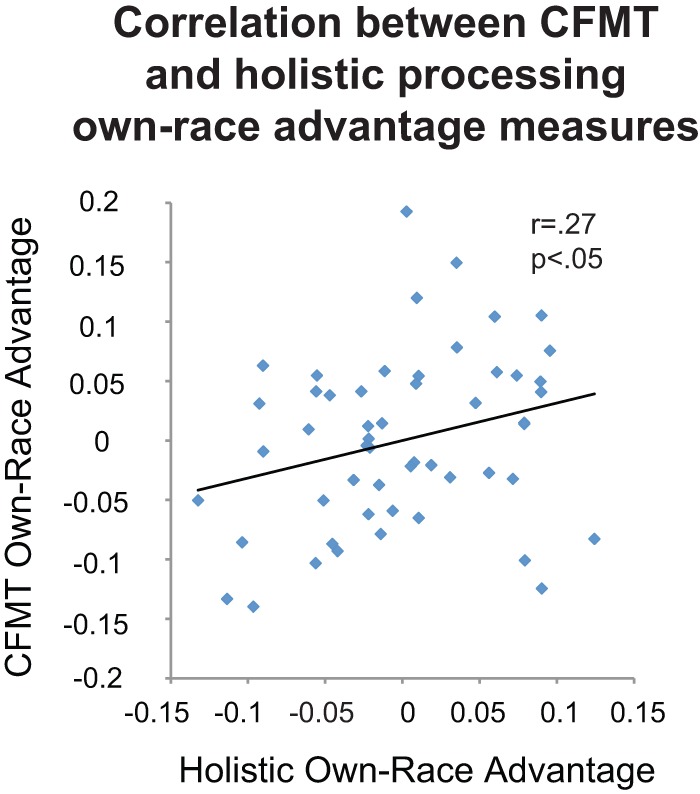
Correlation Between Recognition Memory Own-race Advantage and Holistic Processing Own-race Advantage. *indicates a significant effect p<.05.

In contrast to this subtraction and hybrid subtraction/regression approach, using a regression approach to both measure holistic processing and to separate the ORE into the own-race advantage and the other-race decrement revealed a more robust result. In particular, we found that the greater the boost in holistic processing when one perceives an own-race face compared to an other-race face, the greater the boost in own-race over other-race recognition memory (see Figure 5, r = .27, p<.05). This significant correlation had roughly equal contribution from each feature (eyes: r = .20, p = .16, nose: r = .22, p = .11, mouth: r = .17, p = .24). Considering the reliability of the conditions that went into this calculation and the reduction in reliability from repeated calculations, this overall correlation is approaching its upper bound (CFMT own-race advantage λ2 = .52; holistic processing own-race advantage λ2 = .25; upper bound  =  √(.52×.25) = .36). In comparison, the correlation between the other-race decrement in holistic processing and face recognition was weaker and failed to reach significance (r = .18, p = .20).

After demonstrating a significant association between the own-race advantage in holistic processing and recognition memory, we next sought to determine if this relationship is specific to holistic processing or whether parts-based face processing also showed a similar significant association. Though we did not find a significant difference between own- and other-race faces when analyzing the part trial accuracy (see above), it is still possible that part trial accuracy contributes to the other-race effect in recognition memory (though a somewhat restricted range in differential part accuracy may decrease this correlation). We calculated differential own- and other-race processing for part trials and the CFMT using subtraction, regressing other- from own-race (own-race advantage), and regressing own- from other-race (other-race decrement) and we failed to find any significant associations (ORE subtraction: r = .10, p = .46; own-race advantage: r = .10, p = .48; other-race decrement: r = .15, p = .29). This suggests that, in contrast to holistic processing, differential part processing between own- and other-race faces is not a significant contributor to the recognition memory ORE.

**Does processing own- and other-race faces rely on similar or different mechanisms?** The above results demonstrate enhanced holistic processing of own- compared to other-race faces (particularly of the eye region). Furthermore, individuals who did better on own-race faces than would be expected from other-race performance also had more holistic processing for own-race faces than would be expected from other-race holistic processing performance. Despite these demonstrations that holistic face processing may indeed be an integral component to differential own- and other-race recognition memory, the issue still remains whether own- and other-race face processing rely on similar or different mechanisms. In order to investigate this issue, we first correlated all the individual conditions of the CFMT and PW, as shown in Table 3. The Caucasian and Asian CFMTs were highly associated (r = .79, p<.0001, similar to McKone and colleagues [31]), providing evidence that own- and other-race recognition recruit similar processes. Part trial and whole trial performance was significantly correlated between Caucasian and Asian stimuli (part trials: r = .63, p<.0001; whole trials: r = .72, p<.0001). We also found a significant correlation between Asian and Caucasian holistic processing when using regression to measure holistic processing (r = .37, p<.01) and a weaker relationship when using subtraction to measure holistic processing (r = .24, p = .09). It should be noted that these strong own- and other-race correlations do not undermine the reliable differences we observed between own- and other-race processing, but rather suggest that these differences occur within the context of engaging similar face processing mechanisms.

**Table 3:** Correlations Between Own- and Other-race Recognition Memory and Part-Whole Performance.

	Caucasian CFMT	Caucasian PW_P_	Caucasian PW_W_	Caucasian PW_HP (Reg.)_	Caucasian PW_HP (Sub.)_
Asian CFMT	.79**	.51**	.58**	.35*	0.18
Asian PW_P_	.36*	.63**	.60**	.29*	0.08
Asian PW_W_	.56**	.57**	.72**	.47**	.28*
Asian PW_HP (Reg.)_	.43**	0.22	.43**	.37*	.29*
Asian PW_HP (Sub.)_	0.25	−0.05	0.16	0.23	0.23

We further investigated whether own-race and other-race faces rely on similar mechanisms by measuring if part-whole performance (holistic processing and part trial accuracy) predicts CFMT performance for Asian stimuli to a similar extent to what has been shown with Caucasian faces [24], (see Table 4). For Caucasian faces, we replicated the finding that CFMT accuracy correlates with part-whole part trial accuracy (r = .45, p<.001) as well as holistic processing when computed using regression (r = .47, p<.001) or subtraction (r = .31, p<.05), showing similar results to what was reported in DeGutis et al. [24]. For Asian faces, we also found that CFMT accuracy significantly correlated with part trial accuracy (r = .45, p<.001) and holistic processing when using regression (r = .43, p<.01), although this relationship was only approaching a trend when using subtraction to calculate holistic processing (r = .22, p = .12). This provides evidence that own- and other-race recognition memory comparably rely on holistic and part-based face mechanisms. These findings, along with the robust correlations between Caucasian and Asian CFMTs and PWs, suggest that own- and other-race face processing rely on very similar mechanisms.

**Table 4:** Within-race Correlations of Recognition Memory and Part-Whole Performance.

	Asian CFMT		Caucasian CFMT
Asian PW_P_	.45**	Caucasian PW_P_	.45**
Asian PW_HP_	.62**	Caucasian PW_HP_	.64**

* bold indicates a significant correlation p<.05, ** and bold indicates a significant correlation p<.001.

## Discussion

The current results help to clarify the nature of the other-race effect in recognition memory and its link with holistic processing. First, we replicate previous findings that own-race faces are processed more holistically than other-race faces and further demonstrate that this effect is strongest in the eye region. Also, by using a more valid measure of holistic processing than previous reports and by separating the other-race effect into its component effects (i.e., own-race advantage and other-race decrement), we demonstrated that individuals who recognized own-race faces better than expected given their other-race face recognition also processed own-race faces more holistically than expected given their other-race holistic face processing. Running the same analyses with part trials showed a substantially weaker and non-significant association, suggesting that unlike holistic processing, differential part processing may not substantially contribute to the recognition memory ORE. Finally, for both recognition and holistic processing, performance on own-race faces correlated highly with performance on other-race faces, and for both own-race faces and other-race faces, holistic processing correlated with recognition memory. Together, this suggests that own- and other-race faces recruit similar mechanisms, that own-race faces more thoroughly engage holistic processing, and that this greater engagement of holistic processing is significantly associated with the own-race advantage in recognition memory.

The current results add to the existing evidence of differential holistic processing between own- and other-race faces. Similar to previous studies using the part-whole [6], [8] and composite tasks [7], we found robust differences in holistic processing between own- and other-race faces. We additionally showed that the own- vs. other-race difference in holistic processing was specific to the eye region. This is consistent with demonstrations that the eye region is highly diagnostic for face recognition and may be particularly important for recognizing highly familiar faces [40], [41]. The pronounced difference between own- and other-race holistic eye processing could be because the eye region is the most rich and complicated section of the face and may take the most expertise and/or attentional resources to effectively integrate into a holistic representation [42] (see more on this below). Notably, the current finding of reduced holistic processing of the eye region for other-race faces is quite similar to recent part-whole results in developmental prosopagnosics showing reduced holistic processing of the eye region [43]. Considering these similarities, it is tempting to speculate that these phenomena share a common mechanism. However, this may be an oversimplification as prosopagnosics are also significantly worse at recognizing the eyes and the other features of the face in isolation and not just the eyes within the context of the whole face [43]. What may be common between controls processing other-race faces and prosopagnosics processing faces in general is that the eye region is the most difficult section of the face to process in a holistic manner and may require the most expertise and/or attentional resources [42], which both prosopagnosics and individuals processing other-race faces may lack.

Further demonstrating the importance of holistic processing to the ORE, we also showed that the own-race advantage (i.e., other-race face performance regressed from own-race performance) in holistic processing was significantly linked to the own-race advantage in recognition memory. When considering the modest reliability of the constituent measures (see Table 1), this correlation is near its upper bound, suggesting a robust association. o our knowledge, this is the first time that any aspect of the holistic processing ORE has been associated with any aspect of the recognition memory ORE (Hancock & Rhodes showed this association, but this was confounded by using the same data to measure holistic processing and recognition memory [21]). Considering that the stimuli, timing, and task formats of the part-whole task and CFMT are quite different, these results suggest that this correlation was driven by holistic processing and recognition memory rather than factors such as task format and stimulus similarity. Also, the fact that this correlation was substantially weaker when examining differential own- and other-race face part performance suggests that this relationship is specific to holistic processing. Combined with our demonstration of holistic processing differences between own- and other-race faces, this provides compelling evidence that the degree to which one processes own-race faces in a holistic manner relative to other-race faces is related to the size of the own-race advantage in recognition memory. These findings substantially validate the critical role of holistic processing advocated by current expertise and socio-cognitive models of the ORE [11], [15].

These demonstrations of differential holistic processing of own- and other-race faces and a significant association between the own-race advantage in holistic processing and recognition memory could either occur within the context of own- and other-race faces engaging similar or different mechanisms. We explored this issue by directly correlating our own-race and other-race performance measures and found extremely high correlations between CFMTs (replicating recent work [31]), as well as significant correlations between part trials, whole trials, and holistic advantages in the part-whole task (see Table 3). This suggests that own- and other-race processing engage quite similar mechanisms and that own-race faces engage certain mechanisms, particularly holistic processing of the eye region, more fully. We also found that own- and other-race face recognition are similarly predicted by holistic and part processing PW measures (see Table 4), suggesting that the same perceptual processing mechanisms are related to successful recognition for both own- and other-race faces but that holistic mechanisms are more fully recruited for own-race faces. Along with recent behavioral [31], [44] and eye movement studies [45], this suggests that own- and other-race processing is much more similar than it is different. This calls into question models that propose pronounced processing differences between own- and other-race faces, such as those suggesting that own-race faces are individuated whereas other-race faces are categorized [46]. However, it must be noted that participants were explicitly instructed to individuate all faces in the current tasks, and it is possible that differential own- and other-race processing may occur in more naturalistic contexts.

The current study was not designed to differentiate between expertise and socio-cognitive models of the ORE and instead has important theoretical implications for both approaches. Our findings, along with recent reports [31], [44], suggest that similar mechanisms are used for own- and other-race face recognition. From an expertise perspective, this would suggest that face expertise and the other-race effect accumulate incrementally within the same system rather than involving a marked shift in processing style. Additionally, our demonstration of greater holistic processing of the eye region in own- compared to other-race faces is consistent with the idea that the eye region requires the most expertise to integrate with the rest of the face [6], [42], [43], [47], and suggests that holistic eye processing is the most sensitive to lack of expertise associated with other-race face processing. The finding that the own-race advantage in holistic processing is significantly associated with the own-race advantage in recognition memory emphasizes individual variation in expertise with own-race faces rather than other-race faces. This variation in expertise with own-race faces could be due to genetic factors [32] or differential own-race individuation experience (actively comparing a face to faces in memory) such as from one's profession (e.g., working in isolation as tech support vs. working as a doorman at a busy residential building). These influences may allow some individuals to create a more rich and detailed holistic representation of their own-race facial structure and the variance in that structure across individuals [11] as well as have enhanced recognition memory for own-race faces. This emphasis on own-race individuation is consistent with a recent study suggesting that the act of individuation itself is critical for proficient face recognition [13] and is also consistent with studies showing reductions in the ORE by performing individuation training with other-race faces [5], [13]. According to the current results, it may be that individuals with greater individuation abilities and individuation experiences are able to more fully engage these abilities with own-race faces (demonstrating a relatively larger ORE) and, with enough other-race individuation experience, can also more readily abolish the ORE.

The current results also provide insights to socio-cognitive models. Our demonstration of high correlations between own- and other-race face processing, along with recent studies [18], challenge strong versions of the socio-cognitive categorization-individuation model which suggest different mechanisms are recruited for own- and other-race faces. Instead, the current results suggest a greater emphasis on the individuation advantage for own-race faces rather than the own-race individuation/other-race categorization dichotomy. Additionally, our finding of greater holistic processing of the eyes in own-race compared to other-race faces is consistent with socio-cognitive accounts. Increased attention has shown to enhance holistic face processing [48], and considering that the eye region has the greatest number of elements to be processed holistically (e.g., sclera/iris, eye shape, eyebrows, eye/eyebrow spacing, intraocular spacing, intra-eyebrow spacing, position of eyes/eyebrows on face), it may be most sensitive to changes in attention and holistic processing. Enhanced attention producing greater holistic processing of the eyes could also explain why direct eye gaze (which has shown to capture attention [49]), compared to averted eye gaze, has shown to improve recognition of own-race faces but not other-race faces. It may be that direct eye gaze recruits greater attentional resources for own-race faces compared to other-race faces, which may lead to greater holistic processing of the eye region and enhanced memory for own-race faces. Perceptual expertise accounts have a difficult time explaining this because the pupils/irises are only shifted a millimeter or so between averted and direct gaze conditions, not enough to change the perceptual representation.

When considering the significant correlation between the own-race advantage in recognition memory and the own-race advantage in holistic processing from a socio-cognitive perspective, this result suggests that individuals have some basic level of attention and motivation to process faces of all races and that the ORE reflects enhanced attention when perceiving own-race ‘in-group’ faces. This focus on the own-race advantage and less emphasis on actively categorizing [50] or ignoring [25] other-race faces is in line with recent ORE studies [18] as well as more general in-group/out-group effects in social psychological studies, which predominantly demonstrate a mild form of in-group favoritism rather than out-group derogation [51], [52]. Why might individuals vary in their motivation to attend to in-group members? Recent studies suggest a combination of genetic and environmental factors [53], [54]. For example, individuals have shown to vary in their desire to promote intergroup hierarchies and to have their in-groups to dominate their out-groups (e.g., orientation towards ‘social dominance’) [55]. Future studies that assess the association between inter-group bias measures and the own-race advantage and whether this is mediated by holistic processing would be useful to further characterize these socio-cognitive models of the other-race effect.

When considering the expertise and socio-cognitive accounts of the current results, it should be noted that the 6-face learning format of the CFMT compared to more commonly used ORE old/new paradigms with more numerous faces to learn (e.g., 15 faces) and shorter viewing times may have slightly attenuated the ORE in the current study and made it more dependent on expertise-related mechanisms. For example, more trials of learning with fewer faces may have made subjects more likely to individuate other-race faces than categorize them, potentially reducing the contribution of socio-cognitive factors. However, implicit socio-cognitive attention biases could still play a significant part in the observed CFMT ORE. For example, although there are more opportunities to individuate faces, subjects may not have put as much effort into individuating other-race faces as own-race faces.

In addition to theoretical contributions to both expertise and socio-cognitive models, the current study also provides two clear demonstrations of the methodological utility of regression when studying individual differences. First, we demonstrate that regression can be used to create a more valid measure in cases where there is a clear control condition (e.g., part trials) and a clear condition of interest (e.g., whole trials) (for a review, see Wilmer and colleagues [56]). By regressing part trials from whole trials, this allowed us to create a holistic processing measure that had no relationship with the part trial condition (see DeGutis et al., 2012 for more details [24]). This contrasts the subtraction approach that is typically used to measure the holistic advantage in the part-whole task [39], [57], where holistic processing is confounded by part trial variance. Compared to subtraction measures of holistic processing, regression measures revealed much stronger within-race correlations between holistic processing and recognition memory and a stronger correlation between own- and other-race holistic processing measures.

The current results also demonstrate the usefulness of using regression in individual differences studies when examining the separate components of a bidirectional effect (i.e., where both conditions are of interest and there is no clear-cut control condition). This allowed us to more thoroughly explore the distinct holistic processing/recognition memory relationships of the own-race advantage and other-race decrement. This revealed a significant holistic processing/recognition memory ORE relationship when examining the own-race advantage but not the other-race decrement. No correlation between OREs for holistic processing and recognition memory was observed when subtracting other- from own-race performance. This highlights that subtracting two conditions of interest can obscure theoretically significant relationships. Even if we were to observe a significant association using subtraction, we would have had no insight into how the components of the subtraction measure combined to produce this association. Thus, using regression to separate components of bidirectional effects can more effectively isolate the source of individual difference relationships and provide important theoretical insights.

Though the current study provides several theoretical contributions as well as demonstrating the utility of regression in individual differences studies, one limitation is that we only ran Caucasian participants. That said, we were careful to include only tasks and stimuli that were previously validated using Caucasian and Asian participants and that previously demonstrated significant participant race by stimulus race interactions [8], [31]. Additionally, we reasoned that Caucasian participants would be a better population in which to investigate ORE individual differences since Caucasian participants have shown larger OREs compared to individuals of other races [58]. This would create fewer range restriction issues than with other groups of participants (e.g., Asian participants [7]). A second limitation of the current study is that, because it was part of a larger pre/post training experiment, the tasks were run in a fixed order. Although having a fixed order reduces between-subjects variance due to differences in orders, there could be idiosyncratic effects of the particular order of tasks.

In summary, we present an approach to the other-race effect that allows for its decomposition into the own-race advantage and other-race decrement. In contrast to the lack of a significant correlation when using the traditional subtraction approach to measure the other-race effect, using the regression approach revealed a significant relationship between the own-race advantage in holistic processing and the own-race advantage in recognition memory. This substantially validates current expertise and socio-cognitive theories that emphasize the importance of greater engagement of holistic processing with own-race compared to other-race faces. Our results also show that successful own- and other-race recognition memory depends on very similar mechanisms, suggesting that differential holistic processing occurs within a context of similar own- and other-race processing mechanisms. Together, our findings advocate for a more sophisticated approach to studying the other-race effect in an individual differences context and demonstrate how this approach can bear theoretically important fruit and potentially lead to more nuanced models of this phenomenon.

## Supporting Information

File S1 (DOCX)
