# Supplementary material for: Individual Differences in Holistic Processing Predict the Own-Race Advantage in Recognition Memory
Source: PLoS One. 2013 Apr 10;8(4):e58253. doi: 10.1371/journal.pone.0058253 (PMC3622684; doi:10.1371/journal.pone.0058253)
Supplement: File S1 — (DOCX) [file pone.0058253.s001.docx]

***S1. Supplementary Information***

Methods

*Calculating Reliabilities of measures*

We calculated reliabilities for the Asian and Caucasian CFMT and all the conditions in the Asian and Caucasian PW using Guttman’s λ2 and Cronbach’s α. We chose Guttmans’s λ2 because it is a more robust measure of reliability when the measure includes multiple factors (Callender and Osburn, 1979). The presence of multiple factors is a real possibility in the PW, since it contains multiple trial types (i.e., eyes/nose/mouth trials make up each part and whole condition). These reliability methods showed nearly identical results for the CFMT, but Guttman’s λ2 showed significantly higher reliabilities for the PW conditions, likely because of their multiple trial types. Considering this, in all subsequent analyses we used Guttman’s λ2.

Next, from the reliabilities for the conditions in the Asian and Caucasian PW, we computed the reliabilities of the holistic processing measures using subtraction and regression approaches from equation 1 and equation 2 below, respectively.

Finally, using the resultant reliabilities for the CFMTs, as well as the holistic processing subtraction, holistic processing regression, and part performance for the PW tasks, we calculated the reliabilities of the other-race effect (other-race minus own-race using equation 1), own-race advantage (other-race regressed from own-race using equation 2), and other-race decrement (own-race regressed from other-race using equation 2).

Equation 1:

Reliability of difference scores = {[(rxx + ryy)/2] - rxy} / (1 - rxy)

Where rxx = reliability of condition x; ryy = reliability of condition y; rxy = correlation between condition x and condition y.

Equation 2:

For the regression approach, we used the following equation (Malgady & Colon-Malgady, 1991):

Reliability of residuals = (rxx + rxy² * ryy - 2rxy²)/(1 – rxy²)

Where rxx = reliability of control condition x; ryy = reliability of condition of interest y; rxy = correlation between x and y.

References:

Callender, J. C., & Osburn, H. G. (1979). An empirical comparison of coefficient alpha, Guttman’s Lambda-2, and MSPLIT maximized split-half reliability estimates. *Journal of Educational Measurement, 16*, 89-99.

Malgady, R. G., Colon-Malgady, G. (1991). Comparing the Reliability of Difference Scores and

Residuals in Analysis of Covariance. *Educational and Psychological Measurement, 51*, 803-807.
